# A participatory ergonomics intervention to re-design work and improve the musculoskeletal health of paramedics: protocol for a cluster randomised controlled trial

**DOI:** 10.1186/s12891-023-06834-8

**Published:** 2023-09-08

**Authors:** Karen Davies, Victoria Weale, Jodi Oakman

**Affiliations:** 1 Centre for Ergonomics and Human Factors, School of Psychology and Public Health, La Trobe University, Bundoora, VIC Australia; 2 Queensland Ambulance Service, Brisbane, QLD Australia

**Keywords:** Work-related musculoskeletal disorders, Musculoskeletal pain, Participatory ergonomics, APHIRM toolkit, Paramedics, Ambulance service, Intervention, Cluster RCT, Risk management

## Abstract

**Background:**

In this paper, we present the protocol for a cluster randomised controlled trial to evaluate the effectiveness and implementation of a participative risk management intervention to address work-related musculoskeletal disorders (WMSDs). The aims of the study include to evaluate the implementation process and the impact of the intervention on work related musculoskeletal pain and discomfort and exposure to physical and psychosocial hazards in paramedics over a 12-month period.

**Methods:**

The intervention in this study is to implement A Participative Hazard Identification and Risk Management (APHIRM) toolkit in an ambulance service. Eighteen work groups containing eligible participants (registered paramedics) will be randomised into the intervention or wait-list control arm in one of three rolling recruitment periods. The APHIRM toolkit survey will be offered at baseline and 12 months later, to all current eligible participants in each work group allocated to the trial. The intervention work groups will receive the remainder of the APHIRM toolkit procedures. Identifying data about individual participants will not be collected in the survey, to protect participant privacy and encourage participation. Changes in primary (musculoskeletal pain and discomfort) and secondary (exposure to physical and psychosocial hazards at work) outcomes measured in the survey will be analysed comparing the baseline and follow up response of the cluster. A process evaluation is included to analyse the implementation and associated barriers or facilitators.

**Discussion:**

This study is important in providing a comprehensive approach which focusses on both physical and psychosocial hazards using worker participation, to address WMSDs, a well-known and significant problem for ambulance services. The effectiveness of the intervention in work groups will be rigorously evaluated. If significant positive results are observed, the intervention may be adopted in ambulance services, both nationally and internationally.

**Trial registration:**

ISRCTN77150219. Registered 21 November 2021.

**Supplementary Information:**

The online version contains supplementary material available at 10.1186/s12891-023-06834-8.

## Background

Musculoskeletal disorders are a common and costly health condition affecting 1.71 billion people globally in 2019 [1] which includes workers [2–4]. In Australia, work-related musculoskeletal disorders (WMSDs) have been identified as a leading work health and safety (WHS) problem in terms of frequency and costs, exceeding $24 billion AUD in 2012–13 [5]. Pain and discomfort can be experienced by an individual with a musculoskeletal condition, along with general health problems, anxiety, disturbed sleep and reduced mental wellbeing [3, 6–8], with impacts on absenteeism and disability [9–11]. If work participation is reduced through chronic musculoskeletal conditions such as arthritis or back problems, the cost to the individual and society is significant [12, 13] and this is exacerbated for individuals with other chronic conditions [14]. An extensive evidence base supports the complex aetiology of WMSDs, involving psychosocial, physical and organisational factors [5, 15–17]. However, there is evidence that proactive workplace risk management of psychosocial hazards is not routinely undertaken [18], despite the importance of these in the development of WMSD being highlighted [19–21]. Failure to translate evidence about WMSD risk management into practice was also identified as a gap when Australian organisations undertook investigations related to WMSDs, as a strategy to learn and prevent future incidents [22]. Substantial practical challenges exist in developing, implementing, and measuring workplace interventions to address WMSDs [5], which may explain the current situation.

WMSDs are a major occupational health problem for the healthcare and social assistance workforce [23–28]. In Australia, 8.6 serious claims for workers’ compensation were reported per million hours worked in 2019–20 in the industry [4]. In 2021 there were more than 22,000 paramedics registered in Australia, with approximately equal numbers of males and females, and the majority under 40 years of age [29]. Frontline workers who perform ambulance service work have high exposure to risk factors for WMSD and a high prevalence of adverse musculoskeletal health outcomes related to their work [20, 30–32]. Ambulance officers in Victoria, Australia were 13 times more likely to have a low back workers’ compensation claim compared to nurses during the period 2009—2012 [33]. The critical role of frontline ambulance personnel during the COVID-19 global pandemic and the increasing demand on support services to support their mental health and wellbeing has been recognised [34]. A survey of Australian paramedics about the impact of the COVID-19 pandemic found significant reporting of symptoms of burnout, anxiety and other mental health conditions along with perceived lack of organisational support [35]. Despite the significance of this workforce in the healthcare system and the known problem of exposure to WMSDs, limited research has been undertaken to assess and intervene in the design of work to optimise the musculoskeletal health of ambulance personnel, taking into account the work system within which they operate [30]. Previous research has largely focussed on specific aspects of the role, including stretcher design [31], loading technique [36], paramedic bags [37] and evaluating ambulance patient compartments [38].

WMSDs are complex, outlined in a model by Macdonald and Oakman [17] which refers to three important groups of work-related factors (physical, psychosocial and organisational), and that a poor match between these and individual factors, could lead to hazardous within person effects (high biomechanical load, fatigue, stress response) and increased risk of WMSD in the individual, who may experience discomfort, pain and/or tissue damage [10, 21, 28]. Given this complexity, interventions need to be multilevel and target multiple hazards using principles of systems thinking and implementation science [17, 20]. A multifaceted approach to workplace interventions which addresses tasks, equipment, training, and policy, has been identified as effective in the healthcare and community sector [24, 26, 28] and specifically in patient handling interventions in healthcare settings [25, 39], which were found to be in common use in Australian workplaces [40]. Participatory ergonomics (PE) processes have been identified as important in WMSD prevention [41–43], including in the healthcare sector [42]. A PE intervention has been described as one where workers or end users are involved in the process [44]. The Participatory Ergonomics Framework (PEF) provides a validated set of nine dimensions, with categories to describe the nature of the dimension in a particular intervention and guide the description of PE in practice [45]. Describing PE interventions along with the outcomes they achieve is important, as by the very nature of being participatory they are heterogenous, varying in intensity and implementation process [41, 44]. However, one review identified that many PE studies did not use the PEF to describe the intervention, making comparison of results between studies difficult [41]. A review [44] identified several important factors for successful PE interventions including the right people being involved; addressing barriers/facilitators; defining responsibilities; providing ergonomic training and making decisions using group consultation. Barriers to effective WMSD interventions include failure to adopt a systems approach to risk management; inadequate risk controls; lack of attention to commitment, culture, climate or worker participation; degree of competency to intervene and legislation [5]. Psychosocial factors in the workplace can impact the success of a participative intervention and may be influenced by the intervention itself [6, 43]. For example, poor relationships between workers and managers may hinder the implementation of PE, but a successful PE process may improve the relationships as the PE intervention proceeds over time, providing direct benefits within and indirect benefits beyond the PE process.

This paper details the protocol for a cluster randomised controlled trial (RCT) to evaluate the effectiveness of the implementation of A Participative Hazard Identification and Risk Management (APHIRM) toolkit [46], an evidence based, participative, multifactorial intervention for the prevention of WMSDs, to address the work of paramedics employed in an ambulance service. A limited number of tools are currently available to facilitate a comprehensive assessment of physical and psychosocial hazards in the workplace [47], which may be a barrier to achieving the necessary practice change. The APHIRM toolkit was selected as the intervention for this study because it is freely available and was one of three ‘comprehensive’ tools for risk management of WMSDs in workplaces (that addresses both physical and psychosocial hazards) identified in a recent systematic review [47]. Additionally, the APHIRM toolkit uses a participative approach based on principles of implementation science [46]. The effectiveness of the intervention in work groups, in terms of musculoskeletal health (pain and discomfort) and physical and psychosocial hazards will be measured using quantitative data. A process evaluation will document key activities undertaken in the PE process and identify barriers and facilitators, to inform future interventions using the APHIRM toolkit beyond the outlined trial and addressing the previously identified issue of poor description of PE interventions. A rigorous degree of enquiry is important because high quality studies of workplace interventions have been identified as being needed for some time, to expand the implementation evidence available to organisations wishing to address the problem of WSMDs [25, 26, 28, 41, 48, 49] and potentially accelerate evidence uptake in real world settings.

The aims of this study are to:

1. Evaluate the process of implementing the APHIRM toolkit.
2. Evaluate the impact of the implementation of the APHIRM toolkit on the musculoskeletal health and physical and psychosocial risk profile of work groups, 12 months from initial implementation.

To evaluate the impact of the APHIRM toolkit, the following hypotheses will be tested by comparing the results in intervention and control work groups:

1. There will be a significantly greater reduction in self-rated pain and discomfort scores in the intervention work groups compared to the control work groups over a 12-month period.
2. There will be a significantly greater improvement in the level of self-rated exposure to work-related physical hazards in the intervention work groups compared to the control work groups over a 12-month period.
3. There will be a significantly greater improvement in the level of self-rated exposure to work-related psychosocial hazards in the intervention work groups compared to the control work groups over a 12-month period.

## Methods/design

### Participants

The intervention will be conducted in the Queensland Ambulance Service (QAS), Australia. The organisation employs more than 4,000 ambulance officers, including paramedics, who work from more than 300 ambulance response locations [50] across 1,727,000 square kilometres and servicing more than 5.22 million people [51, 52]. The organisation was approached by the lead author, proposing the APHIRM toolkit as an evidence-based risk management intervention designed to comprehensively address WMSDs. Operational factors may influence the implementation of this study protocol. For example, given the key role of paramedics in the COVID-19 global pandemic and the potential impact on personnel, an increase in COVID-19 case numbers may cause delays to recruitment or the variation of indicative timeframes to move through the intervention. Senior leadership are of critical importance in leading the implementation of APHIRM toolkit; hence, a change in senior leadership personnel could also impact the trial.

### Inclusion and exclusion criteria

To practise in Australia, paramedics must be registered with the Paramedicine Board of Australia [53]. In this study, participants must be registered paramedics, employed and assigned by the organisation for their day-to-day work, to either an intervention or a control work group at the time the survey is offered to that work group. Participants may be of any rank and performing regular direct clinical activities with patients e.g., advanced care paramedics; critical care paramedics; operational supervisors; graduate paramedics. Small, specialised groups of paramedics are excluded from the study to enhance the homogeneity of the potential participant cohort, that is, where their job design is significantly different to the included participants e.g., bicycle response paramedics, flight paramedics. Participants on a continuous period of absence from the assigned workplace and duties, expected to be greater than or equal to five weeks at the time they become eligible for the study, will be excluded because completion of the survey requires recall of the current work exposures. The organisation also employs other ambulance officers who are not registered paramedics, and hosts student paramedics during practical clinical placement. These individuals are not registered, so do not meet the inclusion criteria.

### Intervention

The APHIRM toolkit comprises a set of open source, online tools and procedures designed to guide organisations in planning and taking a comprehensive approach to reduce WMSD risk, using the five stages of the risk management cycle [46]. The APHIRM toolkit focuses on managing the WMSD risk in groups of people doing a particular job, with active participation from workers, addressing three key evidence to practice gaps which have been identified, that is, the typically narrow focus on physical hazards; the need to actively involve workers in the risk management process and that risk controls address risk at the source, in accordance with the hierarchy of controls [46]. The key activities involved in the implementation of the toolkit include conducting an online survey, undertaking a participatory risk management process to develop an action plan, implementation of the action plan and repeating the survey to evaluate the outcomes.

The process of implementing the toolkit will be led by a WHS Practitioner (WHSP) facilitator, the lead author, a Certified Professional Ergonomist, with over 20 years’ experience working in health, safety and ergonomics roles, principally in the healthcare sector. To preserve the fidelity of the implementation of the intervention for this trial and account for available resources, a decision was made to use only one WHSP facilitator. This reduces variability in processes but may impact generalisability of the results to situations where a less skilled or experienced WHSP facilitator is implementing the APHIRM toolkit. The WHSP facilitator is a permanent employee of the organisation, which mimics the ‘real-world’ implementation of the APHIRM toolkit. As a result, they are known to the key management personnel and some individual participants in this study. Some practical benefits arise such as trust and confidence through already having established relationships, which may contribute to successful implementation, but may result in over-estimation of the impact. Also due to familiarity, the WHSP facilitator needs to be cognisant of potential bias and influence on participants. The WHSP facilitator will take active steps to manage any potential conflict of interest throughout the study, including ensuring participants are clear that survey participation is voluntary.

### Survey

The survey is the hazard identification and risk assessment component of the risk management processes. The process of developing the survey is fully described by the APHIRM toolkit authors [46] and information demonstrating the validity of the survey is available [54, 55]. Pilot testing during the APHIRM survey development identified that an online survey would facilitate uptake in workplaces and this was achieved through dedicated software development [46]. During the development of the survey, one study involved administering a pilot survey to a large cohort of paramedics [19, 20].

### Information technology

The APHIRM toolkit portal includes automated survey generation, data analysis and reporting modules. An organisational account, managed by the WHSP facilitator, has been initiated in the APHIRM toolkit portal, following the organisation’s approval of the study. The terms of use for the organisational account include that access to content submitted by the organisation is granted to La Trobe University. The APHIRM toolkit portal holds data collected through the surveys and information generated by intervention work groups and their risk management teams (RMTs). Testing of the portal has been undertaken by the WHSP facilitator on organisational devices (mobile and fixed), to ensure that the APHIRM portal works using a mock RMT, which was used to facilitate engagement of senior management. The portal design provides a 24/7 ‘shop front’ for RMT collaboration and documentation, a vital function in this context where reliance on face-to-face collaboration alone could lead to delays. The organisation also has well established virtual team collaboration tools such as shared team sites and video conferencing available for RMTs in this study and all registered paramedics have access to a tablet device with internet access, while on duty.

### Design of the study

A mixed-methods, two-arm (intervention and wait-list control) cluster RCT will be used to evaluate the implementation and the effectiveness of the APHIRM toolkit in work groups of participants, within the organisation. A work group comprises ideally one, or if needed, more than one, ambulance station. Work groups will be referred to hereafter as a ‘control work group’ or an ‘intervention work group’. The cluster design was selected for several reasons. Firstly, the design reflects the usual way in which an organisation implements WHS initiatives. Typically, the manager of a group of workers, in this case, the Officer-In-Charge (OIC) of an ambulance station, leads an initiative under the guidance of a WHS practitioner. Secondly, a cluster design ensures that all eligible participants in the intervention work group are offered the intervention. This avoids contamination between individual participants which would be expected when randomising individual participants within a work group to intervention or control conditions. The participative process—inherent in the APHIRM toolkit design—means that individual participants within a work group are encouraged to discuss the intervention, thus contaminating results. Lastly, mobility of the workforce between ambulance stations is expected. A study design which relied on comparison of individual survey results 12 months later would be impacted by a substantial drop out rate, compromising the utility of the study. The importance of robust design, conduct and reporting of cluster RCTs is recognised; the study will be conducted in accordance with Consolidated Standards of Reporting Trials (CONSORT) guidelines for RCTs [56].

Due to the availability of resources, with only one WHSP to facilitate the study, a rolling recruitment process is planned, with three consecutive time periods. Three intervention work groups and three control work groups will be randomly allocated, in three batches, to each recruitment period which will commence approximately six weeks apart. The six-week timeframe was deemed to be the minimum time for the initiation and sufficient embedding of the required toolkit components in multiple work groups, following which WHSP facilitator time can be directed toward the next allocation of six work groups. This timeframe will be adjusted to meet the operational requirements of the organisation at the time, if required.

A version of the intervention, taking into account the findings of this study, will be offered to wait-list control work groups at the conclusion of the 12 month follow up survey and study. This design ensures that the workforce feels valued, and the organisation meets its duty of care, while balancing the benefits of rigorous study design, providing an appropriate solution to the ethical issue posed by not offering the intervention to all work groups participating in the study.

A Standard Protocol Items: Recommendations for Interventional Trials (SPIRIT) schedule of enrolment, interventions and assessments for this study has been completed (see Supplementary File 1).

### Primary and secondary outcomes

The primary outcome of this study is self-rated pain and discomfort in five body areas, measured using the APHIRM toolkit survey [46] administered at baseline and 12 months later.

The secondary outcomes of this study are:

1. The level of self-rated exposure to work-related physical hazards, measured using a survey administered at baseline and at 12 months.
2. The level of self-rated exposure to work-related psychosocial hazards, measured using a survey administered at baseline and at 12 months.

### Power and sample size

The cluster design requires the formation of intervention and control work groups containing eligible participants, from which a cluster of participants is then formed. To inform the design of the study, the distribution and number of registered paramedics assigned to stations in the two largest regions in the organisation was reviewed, by examining human resources data. As a result, the mean cluster size (number of expected participants per cluster) for this study was set at 18. This value was then used to calculate the sample size. Whilst all efforts will be made during the study to achieve this mean cluster size, the authors recognise that some clusters will have higher or lower numbers of participants due to a variety of factors, including the size of the work group at commencement and the survey response rate. The number of eligible participants in ambulance stations was found to vary considerably; from a few to more than 40. Excluding small stations from the study or grouping them together to increase the number of eligible participants may seem attractive, however these options required careful consideration. The option to exclude small stations was discarded, to avoid ethical concerns. The option to group small stations will be used to facilitate inclusion, but will be limited, because an intervention led by two managers (OICs) does not mimic the real world and introduces a potential confounding variable. A similar survey was administered with a large cohort of paramedics in Australia and achieved a 38% response rate [19] and a survey of Danish ambulance officers about the physical and psychosocial aspects of their work achieved a 62% response rate [32]. This study is anticipated to result in a high response rate because of the detailed contextualisation to suit the organisation and the current interest of the workforce in WMSDs. The attrition rate was set at 30% based on the response rate of paramedics to similar surveys [19, 32] and a protocol for a workplace intervention study in an emergency service organisation [57]. If the mean cluster size deviates significantly from the estimated value of 18, this will be identified and accounted for in the statistical analysis and the interpretation of results.

Table 1 sets out the sample size and power calculations for primary and secondary outcomes evaluated in the RCT taking into account the cluster design at Alpha = 0.05 and Power = 0.80, ICC = 0.05, mean cluster size = 18 and estimated cluster variation = 0.5625. The results of a previous study that administered an earlier version of the APHIRM survey to a cohort of workers including paramedics were used to estimate the effect size for pain/discomfort, exposure to physical hazards and exposure to psychosocial hazards [20]. A recent study involving high-risk workers, reported a relationship between the physical and the psychosocial hazard profile derived from APHIRM survey responses and the musculoskeletal pain/discomfort scores [54]. A conservative approach was taken by choosing a flat intra-cluster correlation coefficient (ICC) of 0.05. ICCs for outcome variables are generally lower than this [58]. Calculations were done using the software R (v4.0.1) [59], with results cross checked against the National Institutes of Health research methods tools [60] in the case of continuous variables.

**Table 1:** Sample size and power calculations

	Mean	SD	Effect size (d)	Required standard sample size (N)	Required sample size accounting for cluster design (N)	Required sample size after allowing for 30% attrition
Pain/discomfort	14.9	8.3	4	136	317	412
Exposure to physical hazards	3.7	0.6	0.5	46	107	139
Exposure to psychosocial hazards	2.4	0.6	0.5	46	107	139

The required sample size for the intervention arm, accounting for this cluster design was calculated and the sample size was then increased by 30% to allow for attrition at 12 month follow up. Based on the outcome ‘pain/discomfort’ at an effect size of four, we need to recruit a minimum of 18 clusters of 18 or more participants into the trial (*n* = 317/18 = 17.6 clusters; in practice, nine intervention and nine control clusters). For the outcomes ‘exposure to physical hazards’ and ‘exposure to psychosocial hazards’ at an effect size of 0.5, we would recruit a minimum of six clusters of 18 or more participants into the trial (*n* = 107/18 = 5.9 clusters; in practice three intervention and three control clusters). This study aims to recruit 18 clusters (nine intervention and nine control) of 18 or more participants from one region of the organisation to allow for potential attrition.

### Recruitment, randomisation and allocation of work groups

**Fig. 1:**
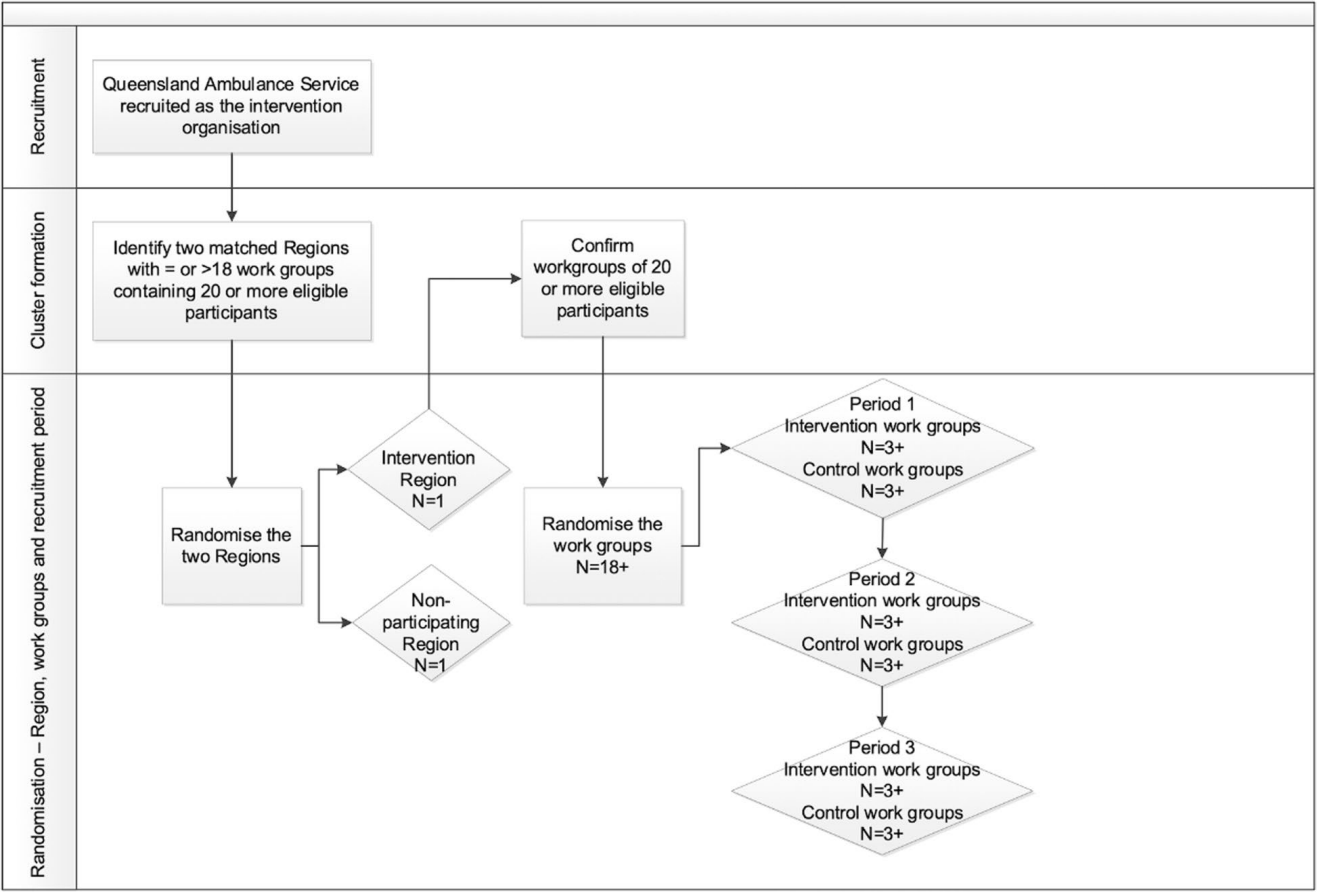
Recruitment and randomisation

The process of recruitment and randomisation is shown in Fig. 1. In October 2021, one of the two identified regions was randomly assigned to act as the intervention region for this study by a blinded employee of the organisation, and the other region discarded from the study altogether. However, due to the ongoing impact of the COVID-19 global pandemic, study commencement was delayed. In early 2022, the situation was re-assessed, and subsequently it was decided that to proceed with the study in a timely manner, the previously discarded region would become the intervention region, to commence recruitment of work groups in June 2022.

Work groups will be finalised as close as possible to the commencement of the intervention. The WHSP facilitator and the region’s senior manager will therefore be aware of the identity of all eligible work groups and participants within them. At this time, work groups will be assigned a de-identified code for randomisation. At the agreed commencement of the first recruitment period, a de-identified list of all eligible work groups will be provided to a research assistant not involved with the study, who will randomly assign six work groups in total to the trial (three to the control and three to the intervention arm) using a random number generator in Microsoft Excel. The research assistant will provide the WHSP facilitator with the de-identified work group allocation for the recruitment period. The WHSP facilitator will re-identify the allocated work groups, thus revealing the allocation immediately prior to the commencement of the intervention. The research assistant will repeat this process at the commencement of the second and third recruitment periods until all eligible work groups have been allocated to the trial.

### Implementation of the APHIRM toolkit

**Fig. 2:**
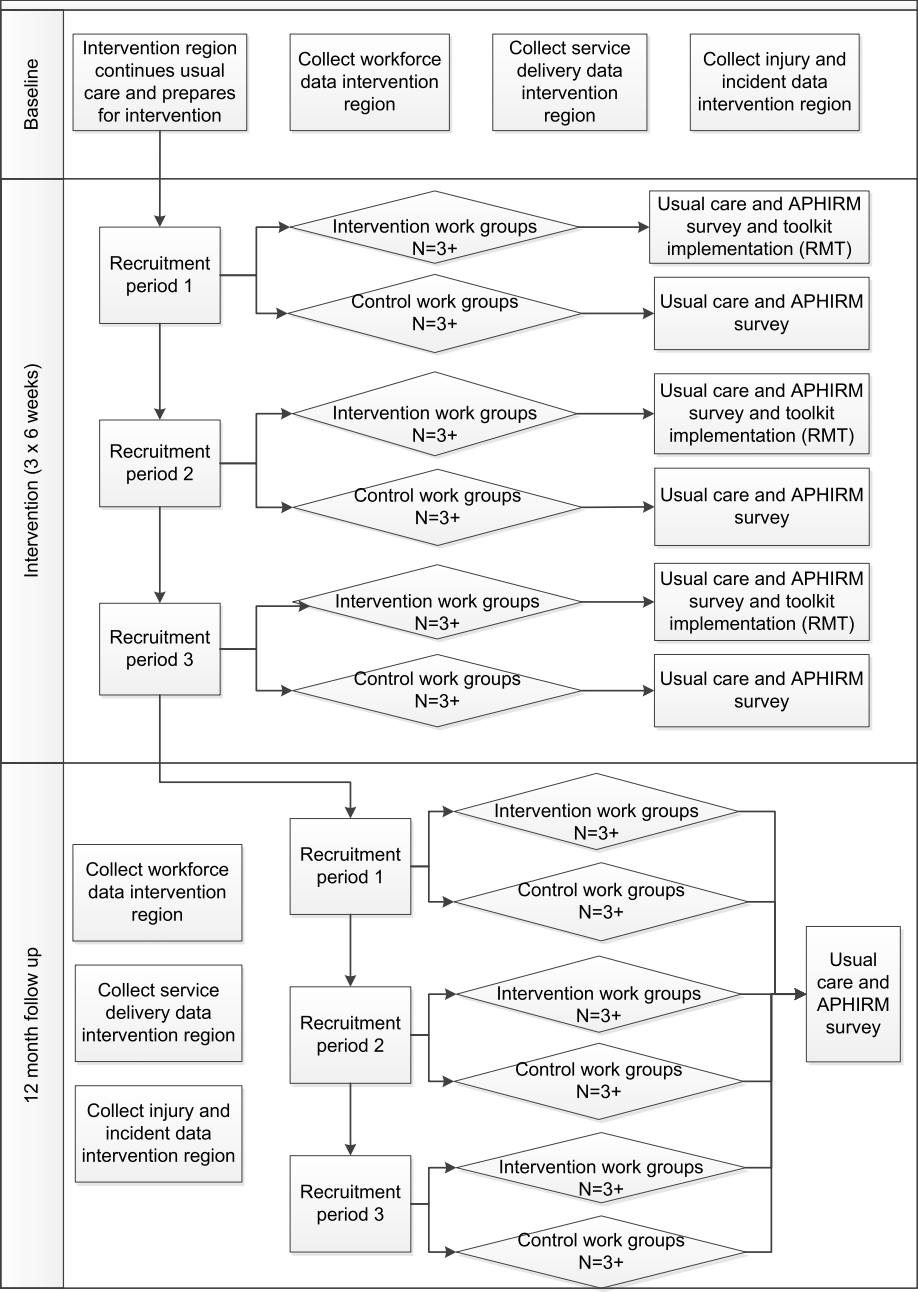
Intervention processes

The processes for the implementation of the APHIRM toolkit in the intervention and wait-list control arms are set out in Fig. 2. The intervention work groups receive the entire APHIRM toolkit process, delivered through the actions of the RMT. That is, the RMT will follow the steps in the toolkit, however, the actual workplace interventions arising from these steps will not be known until the implementation of the toolkit process occurs. In contrast, the wait-list control work groups will only receive the APHIRM toolkit survey at baseline and 12 months afterward. Both groups will continue to receive usual care, that is, the standard WHS management system interventions and practices in the organisation.

### Recruitment of survey participants

Following randomisation into the trial of six work groups, a list of all eligible study participants will be extracted from the organisation’s human resources information system by the approved data custodian. The list will be provided to the WHSP facilitator, who will contact the OIC of each work group and initiate the planning for the survey. Planning includes checking the participant details, agreeing on survey timing and developing communication strategies. The OIC will be advised of their allocation to either intervention or control work group at this time. Although this means the OIC is not blinded to allocation prior to the conduct of the survey, it is considered an important ethical consideration to explain the extent of involvement in the study to the OIC, so that expectations and their personal workload can be managed.

At a time mutually agreed by the OIC and the WHSP facilitator, the APHIRM survey invitation will be sent by email to all eligible participants in the work group. Participants will be invited to complete the online survey via a link in the email to the survey (unique to the work group) with the Participant Information Consent Form (PICF) attached. Participants who submit the survey will be implied to have consented. If participants do not wish to complete the survey, they have the option of skipping all questions. In the organisation, paramedics are provided with an organisational tablet device. Eligible participants who choose to participate will complete the survey during work time on the device. Testing has been undertaken by the organisation to ensure that the survey works on the device and meets security protocols. The survey takes 10–15 min to complete. The identity of individual participants who choose to complete the survey will not be known to the WHSP facilitator, as the survey is anonymous and participation is voluntary. The survey will be open for two weeks, to ensure different shift patterns are captured. However, this timeframe may be extended to meet operational requirements and achieve the required mean cluster size of 18 (survey responses). The WHSP facilitator will be able to monitor the number of responses from a work group during the period the survey is open through the APHIRM portal and provide reminders to the work group and feedback to OICs. OICs will be actively involved in promoting survey uptake, as would be the case in usual practice.

### Formation of risk management team

The next step in the APHIRM toolkit implementation is the formation of a RMT for the intervention work groups. Following closure of the survey, the OIC will be contacted by the WHSP facilitator to discuss membership of the RMT. Suggested members will be typically at least one or two paramedics; the Health and Safety Representative (HSR; if elected); the OIC and a local WHSP, clinical educator or another supervisor. The WHSP facilitator will train the RMT using the resources provided in the APHIRM toolkit, which will be customised to suit the organisation’s operational context. Recruitment to the RMT will therefore not be voluntary, but rather mutually agreed between the OIC and eligible members, replicating the implementation of real world WHS interventions.

### Participatory ergonomics intervention

Following formation of the RMT, the intervention work group will move through the remaining stages of the APHIRM toolkit, considering the hazards and risks identified and developing an action plan. The WHSP facilitator will attend (in person and/or virtually) RMT meetings and support the RMT to follow the participatory processes set out in the APHIRM toolkit. The estimated minimum time to present an action plan to senior management for approval, following the closure of the survey period, is six weeks. However, this timeframe will depend on operational demands. Following management approval of the action plan, the WHSP facilitator will cease to directly support the intervention work group and will move to the next rolling recruitment phase, while remaining available to the RMT for advice and troubleshooting throughout the 12-month study period. The aim is for the OIC to become ‘self-sufficient’ in facilitating the APHIRM toolkit process in their work group. The process evaluation phase of the current study will focus on how and to what degree this is achieved.

### Management of wait list controls

The opportunity for participants in the control work groups to complete the APHIRM survey will be the only activity additional to usual care. OICs will be provided with their work group’s summary of survey results, generated by the APHIRM toolkit software and will be explicitly encouraged to use this in usual care. This will mitigate ethical concerns arising from conducting a hazard identification survey but not conducting the participatory risk management intervention. At the study’s conclusion, the organisation will consider whether the implementation was effective in improving the musculoskeletal health of paramedics and/or their exposure to hazards. Refinements in the process may occur, based on findings from the study in relation to the implementation.

### Data collection

#### Survey measures

The APHIRM survey is the method to collect quantitative data, at baseline and 12 month follow up, in the intervention and wait-list control work groups. The survey firstly confirms a participant belongs to the enrolled work group and is eligible as a registered paramedic (excluding the specialist teams listed previously). Participants are then presented with 54 hazard items covering physical task demands (12 items); physical environment, equipment and occupational health and safety overall (6 items) and psychosocial aspects (36 items). Each item is rated by the participant, using a five-point scale. Four different response scales are utilised. Example items include ‘How much of the time over the last 6 months did you push or pull things, using some force?’ and ‘How often do you find, as part of your work, you have to help people who are upset or unhappy?’, with response options for both items ranging from 1 (Almost never) to 5 (Almost always). A list of the items and the scales in APHIRM is freely available on the APHIRM website [61] and this list was used to engage with the organisation about the survey. Hazard items are grouped in accordance with the Copenhagen Psychosocial Questionnaire [62] categories. There are 14 grouped categories (covering 48 items) and six categories containing an individual item [46]. For example, the item ‘How often do you find, as part of your work, you have to help people who are upset or happy?’ is included in the category ‘Emotional demands’.

Participants rate the frequency and severity of their musculoskeletal pain and discomfort (in the last six months) in each of five body regions: neck/shoulders, arms, hands/fingers, middle/lower back and hips/bottom/legs/feet. For example, ‘In the last six months, how often have you felt discomfort or pain in your neck or shoulders?’ Response options range from 0 (Never) to 4 (Almost always). Any response equal to or greater than 1 (Occasionally) then generates an additional question, for example ‘In the last six months, how bad was the discomfort or pain in your neck or shoulders?’ Response options range from 1 (Mild) to 3 (Severe). A score out of 12 (4 × 3) is calculated for each body region and summed to produce a score out of 60 for each participant reporting any pain/discomfort.

To identify the ‘top 10’ hazards for the RMT work group, Spearman correlations between hazard ratings and individual discomfort/pain scores are calculated in the APHIRM toolkit software [46, 54]. A summary of the survey results for a work group is produced by the APHIRM toolkit software and is provided after survey closure as part of the toolkit procedures.

#### Process evaluation

Data will be collected to enable a process evaluation to be conducted for all intervention work groups. The content of training in the risk management processes and attendance will be recorded. The WHSP facilitator will make field notes following RMT interactions, to record the type of activity, timeframes, who was involved, and subjective impressions of the RMT interactions. For example, the quality of co-operation between RMT members during risk management activities. Data captured in the APHIRM portal about possible solutions, actions and progress of implementation of action plans, provides a detailed description of the course of the implementation of the PE intervention in each work group, for later content analysis. Semi-structured interviews will also be conducted with RMT participants who provide written consent through completing a PICF, to explore their experiences of being part of the RMT. Depending on the number of participants who agree to take part, these may involve RMT members from more than one work group in the same recruitment period. These interviews will be audio taped, transcribed and analysed using NVivo for Windows. The process evaluation will address each stage of the APHIRM toolkit process [46], using a combination of methods as described above. Table 2 sets out the planned process evaluation and data collection plan.

**Table 2:** APHIRM toolkit process evaluation

APHIRM toolkit stage	Process evaluated	Data collection plan
Stage 0 Getting started	• RMT formation/membership • RMT training completed	• WHSP facilitator notes • Training attendance records
Stage 1 Identify main hazards and assess current risk	• Completion of communications • ICT issues reported • Survey response rate	• WHSP facilitator notes • Number of surveys completed/total eligible participants in APHIRM portal
Stage 2 Identify local causes of main hazards	• Degree of match of the risk controls to the hazard • Use of hierarchy of controls • Identification of systems issues outside of local control	• RMT data in APHIRM portal • WHSP facilitator notes
Stage 3 Form action plan	• Action plans completed and on time • RMT function	• RMT data in APHIRM portal • WHSP facilitator notes
Stage 4 Implement action plan	• Evaluation of risk controls completed from action plan • RMT function	• RMT data in APHIRM portal • WHSP facilitator notes
Stage 5 Review	• RMT experience and PE intervention barriers and facilitators	• WHSP conducts semi-structured interviews with RMT who provide written consent to explore PE experience, barriers and facilitators • Thematic analysis using NVIVO

#### Description of service activity, workforce, musculoskeletal injury profile and usual care

Descriptive data including ambulance service delivery, the workforce profile (age; gender; years of service; turnover; absenteeism) and the prevalence of WMSDs (reported incidents and workers’ compensation claims) will be collected at baseline and 12 months later, for the entire intervention region. The OICs of the intervention and control work groups will be invited to complete a short survey at the conclusion of the 12-month period, requesting information about usual care WHS initiatives undertaken during the 12-month study period.

### Analysis

#### Quantitative analysis

The unit for analysis of the primary and secondary outcomes is a cluster (survey responses from a work group). Analysis will be conducted following CONSORT guidelines for cluster-randomised trials [56]. Means for each of the three variables (pain/discomfort score; exposure to physical hazards score and exposure to psychosocial hazards score), at baseline and 12 month follow up, will be generated for each cluster. The mean change by cluster will be calculated by subtracting the baseline from the 12 month follow up score. Analysis will be conducted treating these as continuous variables [63], using a two-sample test, for example a t-test where *n* = total number of clusters, comparing outcomes for the two arms. Data will be cleaned and examined for normal distribution prior to analysis. Differences in cluster sizes will be identified and accounted for using suitable analysis, which will be reported with the results.

The descriptive data will be presented and used when interpreting the results achieved in the work groups. The information collected from wait-list control and intervention work groups about the usual care activities conducted over the 12-month study period, will also provide useful insight to any differences between groups relevant to the analysis.

#### Qualitative analysis

The process evaluation for each intervention work group will be used to determine whether further analyses are needed to explore the influence of the intervention timing, fidelity, or other variables revealed by the semi-structed interviews. During the process evaluation, generated solutions, actions, and progress of implementation will be compared with information contained within the APHIRM toolkit. For example, content analysis will be used to examine congruence between controls arising from the RMT and those suggested in the APHIRM toolkit. Content analysis of solutions, actions, and progress of implementation will also be conducted and compared to best practice. Such analyses will assist in evaluating the degree of effectiveness of the APHIRM toolkit procedures in supporting a more comprehensive approach to WMSD risk management. The thematic analysis of the semi-structured interview data will enable understanding of the RMT members experience during the APHIRM toolkit intervention and the barriers and facilitators of the PE process. This analysis will provide practical insights for organisations considering implementation of the AHPIRM toolkit intervention in the future, to guide optimisation of efforts when resources are constrained.

### Trial status

In principle approval for the study concept was provided by the QAS in August 2020. Study design was then finalised in consultation with the organisation. Ethics approval was granted by the La Trobe University Human Research Ethics Committee in June 2021 (HEC 21166). The randomisation of the control and intervention region was completed in October 2021 and the intervention region revealed to the WHSP facilitator and senior managers in the region, to facilitate briefing and planning. An implementation plan was developed and agreed to by all parties and briefing of senior managers was completed along with testing the technology in the organisation’s operating environment. In late 2021, the trial was paused due to the global COVID-19 pandemic, prior to recruitment and randomisation of the work groups, and in 2022 the situation was reviewed, which led to change in intervention region as described previously. Recruitment commenced in July 2022 and is ongoing.

## Discussion

The prevalence of WMSDs is significant with a high burden on Australian workplaces, including the healthcare sector and with an impact on paramedics [4, 11, 33]. High quality research in relation to interventions to address WMSDs in various healthcare workers groups has been identified as a gap [25, 28, 64]. This study provides a unique opportunity to translate research evidence into practice, to address WMSDs though risk management, using a participatory process in a large ambulance service. This study will inform future dissemination and diffusion of evidence to practice when addressing risk management of WMSDs in paramedics, nationally and internationally. Healthcare organisations may benefit from the insight provided, to guide efforts in addressing the significant problem of WMSDs in the healthcare and social assistance industry. The study will also be of interest to regulators, organisations and WHS practitioners, seeking to understand the processes and outcomes associated with the implementation of the APHIRM toolkit and to apply these in their own context.

The cluster RCT design enables robust analysis of the effectiveness of the toolkit in reducing pain/discomfort and exposure to physical and psychosocial hazards in the workplace. A potential limitation in statistical power to detect clinically significant change in pain/discomfort score is acknowledged. Although the current operating environment in a global pandemic is challenging for a study of this type [34, 35], a rigorous study design and protocol has been developed to control for unexpected variables related to operational context and the ongoing COVID-19 pandemic, while building in flexibility to meet operational requirements. The ability of participants to access the survey on their operational tablet device in work time and limited time required to complete the survey are factors anticipated to significantly contribute to a high response rate to the survey component of the study. A high survey completion rate is critical to ensure that the survey accurately identifies relevant hazards and risks impacting WMSD risk for each work group and sufficient sample size is achieved to determine statistical significance of the results.

The implementation of a participative intervention through the RMT established within intervention work groups is important to document and analyse, as PE processes are known to vary in design and application [41, 43, 44]. For this reason, in addition to the use of quantitative methods to measure the intervention impact on the primary and secondary outcomes, qualitative methods are included to evaluate the implementation process and to explore the experience of key personnel in the process. The use of a mixed methods approach enables the examination and understanding of several steps along the pathway to improved employee health and productivity, for example the barriers and facilitators to the PE intervention, problem identification, solution generation, reduction in risk factor exposures and reduction in pain [41]. Findings will provide valuable insights for organisations considering use of the APHIRM toolkit as an intervention. In addition, the findings will provide insights into the barriers and facilitators of interest to WHSPs, who are typically assigned responsibility to lead the implementation of WHS interventions within organisations.

One key strength of the study design but conversely a practical limitation, is the use of one, highly skilled and experienced internal WHSP facilitator, an ergonomist, to implement the APHIRM toolkit intervention. An alternate scenario in a large organisation is that a senior WHSP facilitator may supervise, coach and support less experienced WHSP facilitators, or work group managers themselves, to distribute expertise, cope with geographical disbursement, manage costs, timeframes and ultimately achieve sustainable change. Future studies could explore whether differences in the results occur where work groups are facilitated by people of varying backgrounds, skills and experience. Caution should be exercised when anticipating what results may be achieved by organisations using different resourcing models and organisational implementation methods, based on the results from this study.

This protocol could be adapted for use in healthcare settings with other patient care occupational groups, nationally and internationally, to further the evidence base about the impact and the implementation of the APHIRM toolkit, supporting further translation of evidence to practice in this large and essential workforce.

### Supplementary Information

**Additional file 1: Supplementary File 1.** SPIRIT schedule.

## Data Availability

Not applicable.

## Abbreviations

APHIRM toolkit: A Participatory Hazard and Risk Management toolkit
HSR: Health and Safety Representative
OIC: Officer-In-Charge
PE: Participatory Ergonomics
PEF: Participatory Ergonomics Framework
QAS: Queensland Ambulance Service
RMT: Risk Management Team
WHS: Work Health and Safety
WHSP: Work Health and Safety Practitioner
WMSD: Work-related Musculoskeletal Disorder
