# Supplementary material for: A participatory ergonomics intervention to re-design work and improve the musculoskeletal health of paramedics: protocol for a cluster randomised controlled trial
Source: BMC Musculoskelet Disord. 2023 Sep 8;24:716. doi: 10.1186/s12891-023-06834-8 (PMC10485987; doi:10.1186/s12891-023-06834-8)
Supplement: Supplementary file 1 — Additional file 1: Supplementary File 1. SPIRIT schedule. [file 12891_2023_6834_MOESM1_ESM.docx]

**Supplementary File 1. SPIRIT schedule**

|  | **STUDY PERIOD** | | | | | | | |
| --- | --- | --- | --- | --- | --- | --- | --- | --- |
|  | **Enrolment** | **Allocation** | **Post-allocation** | | | | | **Close-out** |
| **TIMEPOINT**** | ***0*** | **0** | ***t_1_*** | ***t_2_*** | ***t_3_*** | ***t_4_*** | ***etc.*** | ***t+12 months*** |
| **ENROLMENT:** |  |  |  |  |  |  |  |  |
| **Eligibility screen** | X |  |  |  |  |  |  |  |
| **Informed consent** |  |  | X | X | X |  |  | X |
| **Allocation** |  | X |  |  |  |  |  |  |
| **INTERVENTIONS:** |  |  |  |  |  |  |  |  |
| ***Participatory ergonomics t_1_*** |  |  | X |  |  |  |  | X |
| ***Participatory ergonomics t_2_*** |  |  |  | X |  |  |  | X |
| ***Participatory ergonomics t_3_*** |  |  |  |  | X |  |  | X |
| **ASSESSMENTS:** |  |  |  |  |  |  |  |  |
| ***Service activity, workforce and musculoskeletal injury profile*** | X |  |  |  |  |  |  | X |
| ***Pain/discomfort*** |  |  | X | X | X |  |  | X |
| ***Exposure to physical hazards*** |  |  | X | X | X |  |  | X |
| ***Exposure to psychosocial hazards*** |  |  | X | X | X |  |  | X |
